# Dynamics of emotional disorders in students of medical university in the context of the covid-19 pandemic

**DOI:** 10.1192/j.eurpsy.2022.1819

**Published:** 2022-09-01

**Authors:** T. Mozgova, I. Leshchyna, I. Tieroshyna, H. Kozhyna, S. Fedorchenko, L. Gaichuk, K. Zelenska, I. Strelnikova

**Affiliations:** 1 KNMU, Psychiatry, Kharkiv, Ukraine; 2 KNMU, Neurology, Kharkiv, Ukraine

**Keywords:** Students of Medical Univercity

## Abstract

**Introduction:**

The covid-19 pandemic is having a significant impact on the mental health of the entire population.

**Objectives:**

To determine the dynamics of emotional disorders in medical students in the context of the covid-19 pandemic.

**Methods:**

**Methods of research.** An online survey of medical university students was conducted during the covid-19 pandemic. The Beck scale was used.

**Results:**

**Results and its discussion.** According to a dynamic study of emotional disorders in medical students, which was held during 12 months of covid-19 pandemic, emotional disturbance in the form of depressive manifestations associated with the covid-19 pandemic did not reduced. In addition, the results of the study indicated a change in the structure of depressive manifestations in the surveyed in favor of milder depressive manifestations (27.2%; 24.0%) and a decrease in the prevalence of moderate and severe manifestations of depression (3.0%; 5.3%).

**Conclusions:**

The long-covid pandemic has a negative effect on the mental health of medical students and lead to emotional disturbances in the form of depressive manifestations of varying severity. The compensatory possibilities of mental activity proceed unilaterally with a change in the structure of emotional disorders; adaptation to a stress factor is not formed. Disclosure of interest.– The authors have not supplied a conflict of interest statement.

**Disclosure:**

No significant relationships.

